# Effects of Axial Traction during Direct MR-Arthrography of the Wrist in Sports Injuries

**DOI:** 10.5334/jbr-btr.918

**Published:** 2016-07-29

**Authors:** Benjamin Dallaudière, Marie-Hélène Moreau-Durieux, Ahmed Larbi, Anne Perozziello, Pascal Huot, Philippe Meyer, Lionel Pesquer

**Affiliations:** 1 Centre Imagerie Ostéo-articulaire, Clinique du Sport de Bordeaux, 2, rue Nègrevergne, 33700 Mérignac; FR

**Keywords:** MRI, traction, wrist, ligament, injury, sports

## Abstract

**Purpose:**

To evaluate the degree of joint distraction during direct MR arthrography with axial traction in sports injuries. To confirm the effect of axial traction on the quality of wrist opacification.

**Patients and methods:**

Seventeen patients (11 male, 6 female) underwent wrist MR arthrography without (mean: 39 years (SD 11.6))., and 20 patients (16 male, 4 female) with axial traction (mean: 28 years (SD 7.1)). Subgroups were defined according to pathology: degenerative, ligamentous, traumatic, normal (ie patients without MR-arthrography lesions). Radioscaphoid, radiolunate, lunocapitate, ulna Triangular Fibrocartilage (TFC), scapho-lunate, luno-triquetral, ulnocarpal, Carpo-Metacarpal (CMC) I and III and distal radio-ulnar spaces were measured for all patients. Differences in joint space width were compared between subgroups. Joint space opacification was subjectively scored from 0 (no opacification) to 3 (fully opacified), and compared between the groups with and without traction.

**Results:**

The difference in joint space was statistically significant (*p*<0.05) for radioscaphoid, radiolunate, lunocapitate and ulnocarpal spaces, but only in patients with ligamentous tears. Opacification score was significantly higher for ulnocarpal (*p*=0.0275) and CMC III joint spaces (*p*=0.0272) with axial traction.

**Conclusion:**

Axial traction resulted in a significantly higher radioscaphoid, radio-lunate, lunocapitate and ulnocarpal joint spaces width. This positive effect of axial traction raises the suspicion of sports ligamentous lesions.

## Introduction

In the general population, sports related joint injury occurs most often on the knee (24.7%) and ankle (18.8%), whereas elbow and wrist together account for about 5.5 per cent [1]. Among athletes, between 3 and 9 per cent of all sports injuries involve the hand or the wrist [2]. On the wrist, high impact sports such as auto racing, football, or alpine skiing will more often cause displaced fractures, dislocations, ligamentous and tendon tears. On the other hand, low impact sports like tennis, golf and basketball will more likely be responsible for nondisplaced fractures, contusions, stress reactions, ligamentous sprain or tenosynovitis [3]. In the setting of sports-related wrist injury, the scaphoid is the most commonly fractured carpal bone, whereas ligamentous tears will occur mostly at the scapholunate ligament. In both cases, the injury mechanism is a fall on an outstretched hand. Unlike bone fractures however, ligamentous lesions are often initially missed [2].

Conventional radiography has only a sensitivity of 57 per cent and a specificity of 98 per cent in the diagnosis of scapholunate ligament tears. When using dynamic cineradiography, the sensitivity increases to 85 per cent and specificity decreases slightly to 95 per cent [4]. In a recent cadaveric study, direct multidetector Computed Tomography (CT), Computed Tomography arthrography (CTA) and Magnetic Resonance arthrography (MRA) have respective sensitivity and specificity of 100/100 and 100/86 for the detection of scapholunate ligament tears, which compares favourably to the 66 per cent sensitivity and 86 per cent specificity reported for 3T-MRI without intra-articular contrast media administration, using arthroscopy as golden standard [5]. In another study, the limited value of conventional MRI in the diagnosis of partial intrinsic ligament tears was shown [6].

Despite its superior contrast resolution, MRA seems to be less sensitive than CTA in the detection of partial tears of the scapholunate and lunotriquetral ligaments, partial-thickness tears to the triangular fibrocartilaginous complex and cartilage abnormalities [7].

In several large joints (shoulder, hip or knee), the benefit in term of accuracy and opacification of axial traction towards joint opacification during direct MR arthrography has been shown [8,9,10]. Moreover, a few recent publications have explored the potential benefit of adding axial traction to the wrist during direct MR arthrography [11,12,13]. As shown in a feasibility study by Guntern et al., a 3 kg axial traction to the wrist during the MR scan can improve the exposure of the articular cartilage to contrast medium, increase radio-carpal and lunocapitate joint spaces width [11]. In another study by Cerny et al, the number of detected partial and complete scapholunar (SL) and lunotriquetral (LT) tears increased with traction, but did not reach statistical significance [12]. To our knowledge however, a subdivision of the patient populations in function of the underlying wrist pathology was not made in these earlier publications. Therefore, in this study, we aimed to further evaluate the influence of a broad range of wrist pathologies (traumatic, degenerative, ligamentous) on the degree of joint distraction. A secondary objective was to evaluate the effect of traction on the quality of joint space opacification.

## Patients and Methods

### Study population

The study population consisted of 37 consecutive patients who were referred to our imaging department by an orthopaedic surgeon, with the clinical suspicion of sports related wrist injury from September 2012 to December 2013. They were randomly split into two groups: 17 patients underwent MRI without traction (group 1), and 20 were scanned using axial traction (group 2). Exclusion criteria included absolute and relative contraindications to MRI. All patients were informed of the study procedure and gave informed consent.

### Technique: Wrist arthrography and MRI

**Figure 1:**
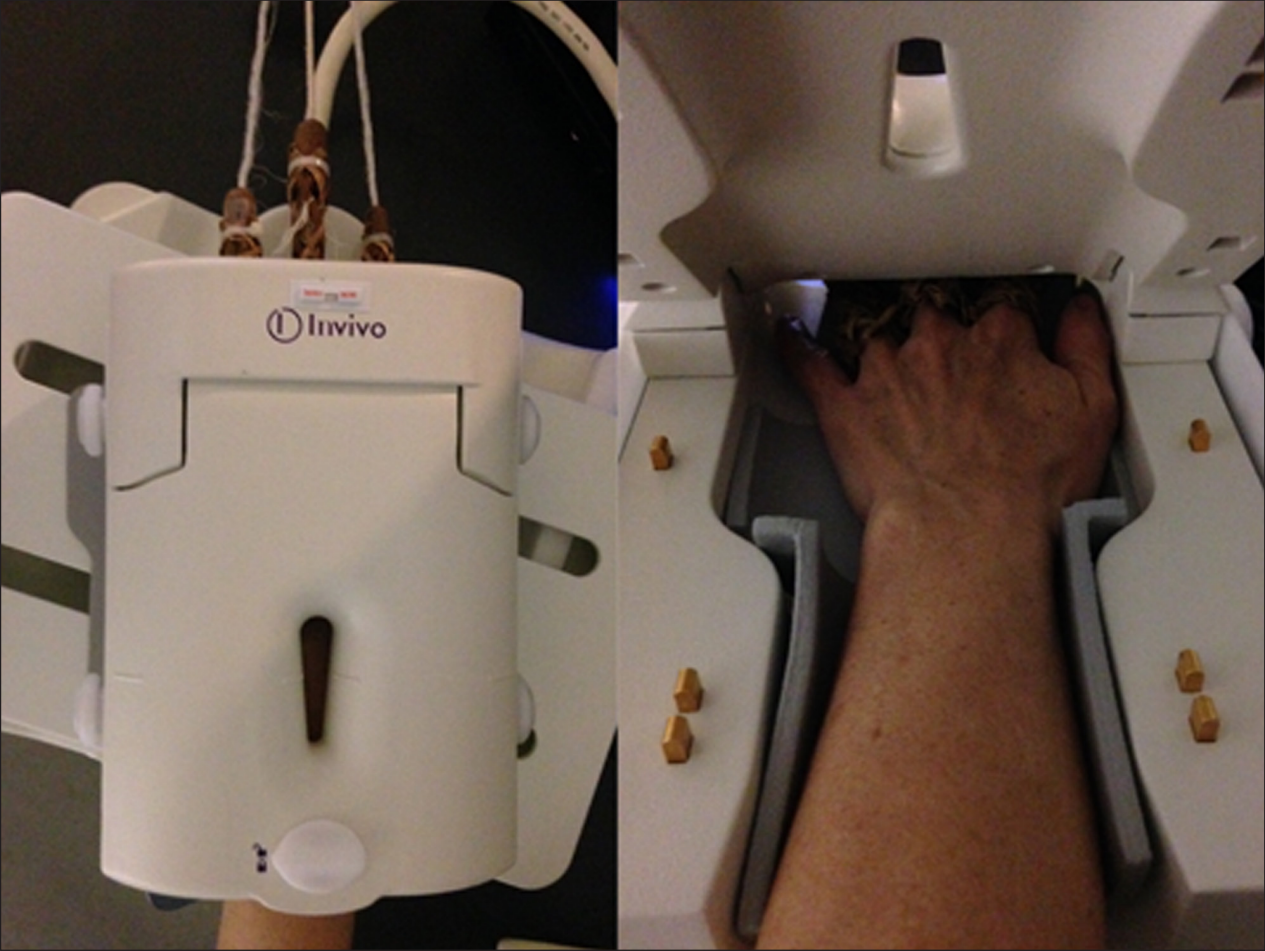
Outside and inside view of the wrist during wrist MRI in traction.

In all patients, a three-compartment wrist arthrography was performed under fluoroscopic guidance using a 23 G needle and a Gadolinium based solution (Artirem® – Guerbet, France). The following blend was used: 0.1 mL of Gadolinium diluted in 10 mL of saline, along with 5 mL of 1 per cent Lidocaine and 5 mL of iodinated contrast agent (Hexabrix® – Guerbet, France) to enable fluoroscopic visualization. The solution was injected into the midcarpal joint at the triquetrohamate space. Subsequently the radiocarpal joint was injected at the radioscaphoid space, and finally the distal radioulnar joint [14]. We mobilized the wrist after injecting every single joint space to detect subtle communications between the different joints. When injection in a given compartment resulted in the opacification of a neighbouring space, this latter compartment was not injected separately. A visually satisfactory opacification of these three spaces was achieved in all cases and no patients reported any major discomfort during this stage. Immediately after arthrography, patients were transferred to the MR suite. MRA was performed with a 1.5Tesla MR scanner unit (Twin Speed HDX: GE® healthcare, MWK, USA). The patients were put in a prone position, with the arm in overhead extension and pronation, An eight channel high resolution dedicated wrist coil was used. In the traction group, a weight of 3 kg on a wire was attached to the hand (finger number two to four including the long finger) using Chinese finger traps and then hung over the distal edge of the scanner table, hereby creating a pulley mechanism that exerts a force on the wrist in an axial direction. Chinese finger traps are small cylindrical cuffs designed to tighten around the fingers when stretched outward (Figure 1). The weight applied in this study corresponds to earlier publications on MRA [11,12] and can be considered safe. We believe three fingers should be trapped to ensure optimal repartition of the weight, and that manual traction should be exerted on the wrist right before attaching the weights and locking the coil, to ensure adequate joint distraction [11,12,15].

All patients underwent an MRA protocol that is based on our own experience [16,17,18]: axial and sagittal T1SE with fat saturation, coronal T1SE without fat saturation, and axial or coronal PDFS. T1-weighted images both with and without FS are included to enable differentiation between Gadolinium and fat. The fluid-sensitive sequences (PD) are included to search for bone marrow edema (Table 1).

**Table 1:** Wrist MRI technical characteristics.

	Coronal T1	Axial T1 FS	Sagittal T1 FS	Coronal DP FS	Axial DP FS

FOV	12	10	12	12	12
Slide (mm)	2.8	3	3	2.8	2.8
Gap (mm)	0.3	0.3	0.3	0.3	0.3
Frequency	384	320	384	416	416
Phase	320	288	320	320	320
Voxel (mm3)	0.328	0.324	0.351	0.302	0.302

### Image interpretation

The evaluation of the MR scans was performed by consensus and in random order by two radiologists with five and two years of experience in the field of musculoskeletal imaging. They were blinded to the clinical data and the presence of traction. For imaging interpretation, a commercially available Picture Archiving and Communication System (PACS) was used (Fujifilm®, USA). The quality of joint opacification was assessed using the semi-quantitative methods described in semi-quantitative analysis paragraph. Based on the MR findings, patients in the two groups were further classified into the following subgroups: degenerative, ligamentous, traumatic, normal. The degenerative group included degenerative cartilage lesions whereas the traumatic group and ligamentous group included bone lesions such as fracture and ligament tears respectively. The normal group was only based on imaging as all patients had symptoms indicating arthrography. There were no healthy persons for comparison.

The joint space width was assessed using the quantitative method, described in quantitative analysis paragraph. All the semi-quantitative and quantitative measurements were made on coronal T1SE without fat saturation.

### Semi-quantitative analysis

The quality of opacification was assessed at the radioscaphoid, radiolunate, luno-capital, ulnar-TFC, SL, LT, ulnocarpal, carpometacarpal (CMC) I and III, and distal radio-ulnar spaces, based on a four-point scale: 0 = no opacification, 1 = less than 50 per cent coverage, 2 = more than 50 per cent coverage, 3 = complete coverage of the articular surfaces with contrast medium on all slices [11,12].

### Quantitative analysis

For the quantitative analysis, the minimum joint space width was measured for the same joint spaces.

**Figure 2:**
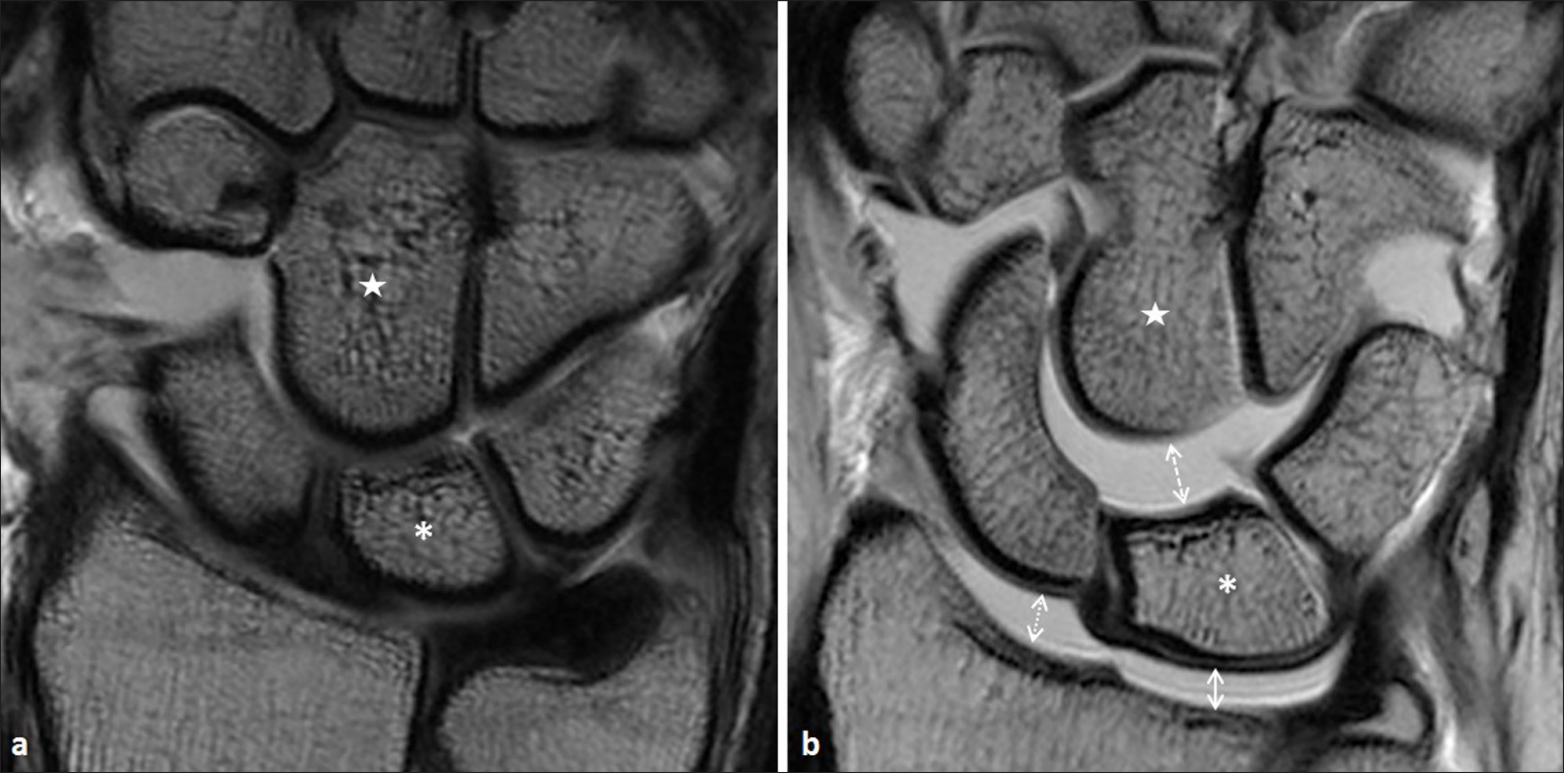
T1-weighted image in the coronal plane in two asymptomatic patients, after intra-articular injection of Gadolinium. Lunate bone is marked with an asterisk, capitatum with a star. **a.** Without traction. **b.** During axial traction, in a wrist well responding to traction. There is a marked widening of the radiolunate (*white full double arrow*), radioscaphoid (*white dotted double arrow*) and lunocapitate (*white dashed double arrow*) spaces.

The shortest distance on any coronal slice between opposing bone structures was used where appropriate. For the SL and LT spaces, the midpoint between Gilula’s first and second carpal arcs was taken as a reference point, since this method has been used before in the literature [11,12]. The same reference article was used to determine the ulna-TFC space: the shortest distance between the cortex of the ulnar head and the nearest border of the TFC. Correspondingly, the luno-TFC space was defined as the shortest distance between the lunate cortex and the nearest border of the TFC (Figure 2).

### Statistical analysis

Statistical analysis was performed using SAS Software (SAS Institute, Inc., Cary, North Carolina). Means concerning quality of opacification and joint space width were compared using the Kruskall-Wallis test (i.e. one-way ANOVA test). A *p* value of <0.05 was considered statistically significant.

## Results

### Population

A total of 27 male and 10 female patients were included in the study (mean age 34 years, standard deviation 11.1). In the traction group, mean age was 28 years (SD 7.1). In the group without traction, mean age was 39 years (SD 11.6).

The age and sex distribution for the different subgroups is shown in Table 2. There was no difference (*p*=0.29) in gender but a significant difference in age (*p*=0.003). Twenty-four patients had three compartmental injections and three had only two mediocarpal and radiocarpal injections (two SL ligament tear and one LT ligament tear).

**Table 2:** Age and sex distribution for the different subgroups.

	degenerative		ligamentous		Traumatic		normal	

	Witd traction (*n*=4)	w/o traction (*n*=4)	Witd traction (*n*=9)	w/o traction (*n*=5)	Witd traction (*n*=4)	w/o traction (*n*=4)	Witd traction (*n*=3)	w/o traction (*n*=4)

Mean age (*StDev*)	40 (6.94)	31 (7.36)	40 (12.33)	34 (6.53)	34 (7.7)	24 (4.7)	40 (16)	23 (2.9)
Male/Female	3/1	3/1	8/1	3/2	3/1	4/0	2/1	1/3

StDev = standard deviation. w/o = without.

**Figure 3:**
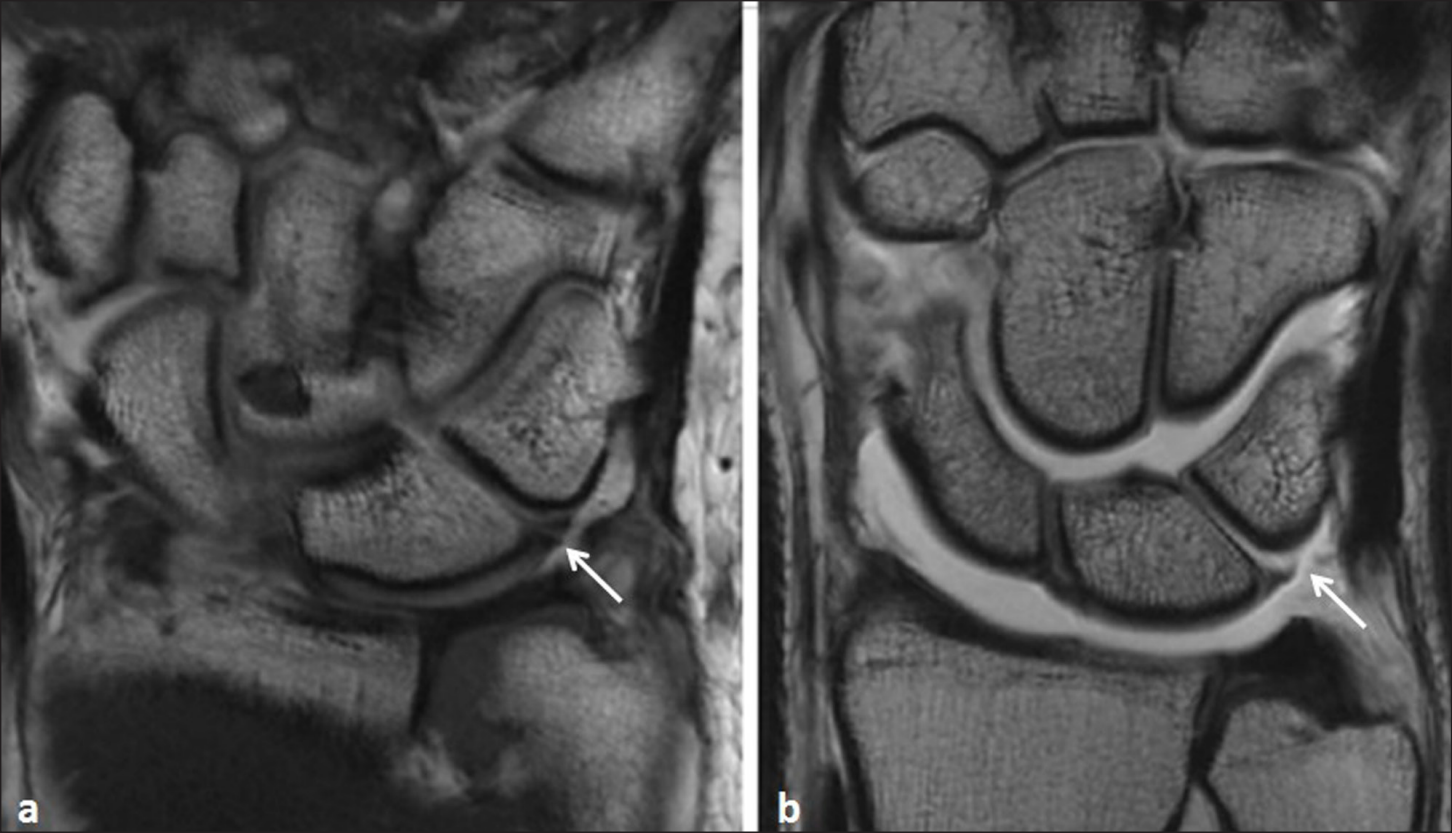
T1-weighted images in the coronal plane in two symptomatic patients with a lunotriquetral ligament tear. **a.** Without traction. The tear is visible as a thin interruption of the ligament (*arrow*). **b.** With traction. In this case, the tear is distended by contrast medium (*arrow*).

**Figure 4:**
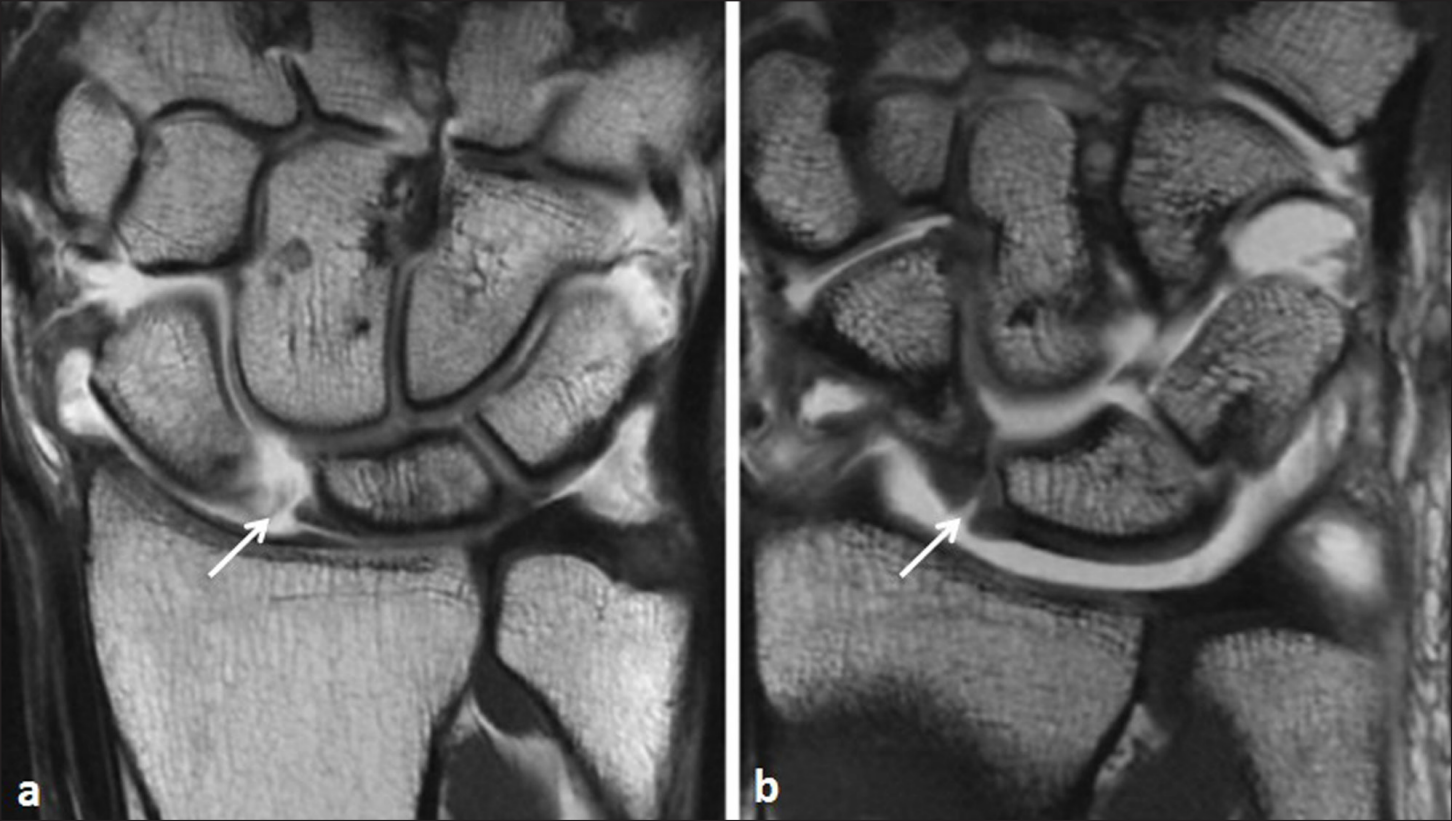
T1-weighted coronal images in two symptomatic patients with a scapholunate ligament tear. **a.** Without traction. There is marked widening of the scapholunar interval (*arrow*). **b.** With traction. Apart from the tear (*arrow*), there seems to be a step-off deformity in Gilula’s most proximal arc.

In the ligamentous subgroup, clinical diagnosis was TFC tear (*n*=5 (three complete tears and two partial tears)), SL ligament tear (*n*=5 (three complete tears and two partial tears)), LT ligament tear (*n*=3 (two complete tears and one partial tear)) (Figure 3 and 4). In the degenerative subgroup, clinical diagnosis was cartilage fissure (*n*=4), arthrosynovial cyst (*n*=3), extensor carpi ulnaris tenosynovitis (*n*=3), flexor and extensor tenosynovitis (*n*=1). In the traumatic subgroup, clinical diagnosis was scaphoid pseudartrosis (*n*=5), bone contusion (*n*=2), hamate fracture (*n*=1).

### Semi-quantitative analysis

Table 3 summarizes the semi-quantitative opacification scores for all joint spaces in the traction group. For the ulnocarpal (*p*=0.0275) and CMC III (*p*=0.0272) spaces, there was a significant difference in opacification score between traction and no traction, all subgroups confounded. For the ulnocarpal space, mean opacification scores and SD were, respectively, 1.00 and 0.15 (degenerative subgroup), 2.60 and 0.90 (ligamentous subgroup), 2.50 and 0.58 (traumatic subgroup) and 3.00 and 0 (normal subgroup). For the CMC III space, mean opacification scores and SD were, respectively, 1.75 and 0.96 (degenerative subgroup), 3.00 and 0 (ligamentous subgroup), 1.75 and 0.96 (traumatic subgroup) and 2.50 and 0.58 (normal subgroup).

**Table 3:** First four columns: Opacification scores for the different joint spaces and pathologies, all with traction (degenerative *n*=4, ligamentous *n*=9, traumatic *n*=4 and normal *n*=3). Right column: *p* values, traction vs no traction for all joint spaces, all pathologies confounded.

	degenerative	ligamentous	traumatic	normal	*p*

Radioscaphoid	2.25 (*0.96*)	3.00 (*0*)	2.75 (*0.5*)	3.00 (*0*)	0.18
Radiolunate	2.25 (*0.96*)	3.00 (*0*)	3.00 (*0*)	3.00 (*0*)	0.0749
Lunocapitate	2.25 (*0.96*)	3.00 (*0*)	2.00 (*0.82*)	3.00 (*0*)	0.0523
Ulna-TFC	1.50 (*1.29*)	1.00 (*1.41*)	0.25 (*0.5*)	0 (*0*)	0.1731
Scapholunate	2.25 (*0.96*)	3.00 (*0*)	2.25 (*0.5*)	1.75 (*0.96*)	0.1038
Luno-triquetral	1.75 (*0.5*)	2.20 (*0.84*)	1.25 (*0.5*)	2.00 (*0.82*)	0.2546
Ulnocarpal	1.00 (*1.15*)	2.60 (*0.90*)	2.50 (*0.58*)	3.00 (*0*)	0.0275
CMC I	2.00 (*0.82*)	0.60 (*0.89*)	0 (*0*)	0.50 (*0.58*)	0.0711
CMC III	1.75 (*0.96*)	3.00 (*0*)	1.75 (*0.96*)	2.50 (*0.58*)	0.0272
DRUJ	0.75 (*1.5*)	0.60 (*1.35*)	0 (*0*)	0 (*0*)	0.5835

### Quantitative analysis

The results of the quantitative measurements are shown in Table 4. All the significant results fell within the ligamentous subgroup: the radioscaphoid space increased from 1.28 mm at rest to 3.88 mm during traction (*p*=0.0116); the radiolunate space increased from 1.15 mm to 2.98 mm (*p*=0.0116); the lunocapitate space increased from 1.30 mm to 2.81 mm (*p*=0.0193); the ulnocarpal space increased from 1.47 mm to 2.81 mm (*p*=0.025).

**Table 4:** Measured joint space width for the different spaces (mean values in millimeters).

	degenerative			ligamentous			traumatic			normal		

	mean (*StDev*)		*p*	mean (*StDev*)		*p*	mean (*StDev*)		*p*	mean (*StDev*)		*p*
	traction = 0 (*n*=4)	traction = 1 (*n*=4)		traction = 0 (*n*=9)	traction = 1 (*n*=5)		traction = 0 (*n*=4)	traction = 1 (*n*=4)		traction = 0 (*n*=3)	traction = 1 (*n*= 4)	

Radioscaphoid	2.09 (*1.19*)	1.59 (*0.70*)	0.678	1.28 (*0.46*)	3.88 (*0.60*)	0.0116	1.47 (*0.23*)	3.54 (*0.89*)	0.0671	1.16 (*0.35*)	1.96 (*1.23*)	0.6149
Radiolunate	1.46 (*0.66*)	1.53 (*0.34*)	0.8893	1.15 (*0.50*)	2.98 (*0.50*)	0.0116	1.39 (*0.30*)	2.61 (*0.81*)	0.0658	1.64 (*0.97*)	2.24 (*1.34*)	0.6149
Lunocapitate	1.24 (*0.55*)	1.15 (*0.31*)	/	1.30 (*0.21*)	2.81 (*0.90*)	0.0193	0.86 (*0.24*)	2.63 (*2.05*)	0.0671	1.02 (*0.31*)	2.12 (*1.69*)	0.8655
Ulna-TFC	0.77 (*0.55*)	1.40 (*0.79*)	0.4939	0.58 (*0.40*)	1.00 (*0.36*)	0.1332	0.44 (*0.30*)	0.61 (*0.41*)	0.2835	0.57 (*0.38*)	0.35 (*0.40*)	0.6118
Scapholunate	1.38 (*0.59*)	1.29 (*0.22*)	/	1.80 (*1.87*)	1.28 (*0.45*)	0.7939	1.02 (*0.16*)	1.64 (*0.31*)	0.0816	0.89 (*0.32*)	1.31 (*0.23*)	0.1247
Luno-triquetral	0.97 (*0.29*)	0.90 (*0.29*)	0.8893	0.74 (*0.27*)	0.82 (*0.25*)	0.6953	1.00 (*0.31*)	0.74 (*0.22*)	0.346	0.77 (*0.45*)	0.74 (*0.23*)	/
Ulnocarpal	1.28 (*0.22*)	1.10 (*0.79*)	0.8893	1.47 (*0.49*)	2.81 (*0.83*)	0.025	1.10 (*0.31*)	1.90 (*0.26*)	0.0671	1.54 (*0.28*)	2.42 (*1.26*)	0.4108
CMC I	0.98 (*0.47*)	1.06 (*0.34*)	0.8893	1.18 (*0.45*)	1.05 (*0.35*)	0.7939	1.05 (*0.45*)	1.53 (*0.35*)	0.1564	1.58 (*0.35*)	1.19 (*0.28*)	0.2622
CMC III	0.61 (*0.19*)	0.63 (*0.16*)	/	0.58 (*0.13*)	0.98 (*0.47*)	0.1336	0.62 (*0.27*)	1.02 (*0.13*)	0.1564	0.74 (*0.50*)	0.95 (*0.34*)	0.6118
DRUJ	0.75 (*0.26*)	0.94 (*0.35*)	0.678	0.83 (*0.22*)	1.08 (*0.42*)	0.3671	0.78 (*0.40*)	1.30 (*0.43*)	0.346	0.91 (*0.52*)	0.63 (*0.17*)	0.6149

The four columns represent different pathological subgroups. *TFC = triangular fibro-cartilage. CMC = carpometacarpal. DRUJ = distal radio-ulnar joint*.

## Discussion

In the present study, opacification score was significantly better for ulnocarpal and CMC III joint spaces with axial traction. Moreover, a significant increase in joint width during traction was observed for the radiocarpal (radioscaphoid, radiolunate, lunocapitate) and ulnocarpal spaces, but only in the subgroup with ligamentous injury. In our opinion, this might be related to the sports ligamentous pathology in these patients.

Guntern et al were among the first to investigate the effect of axial traction during MRA. They observed a significant widening of the radioscaphoid, radiolunate, and lunocapitate spaces, with corresponding improvement of opacification, results that are comparable to ours [11]. In their study however, the possible effect of underlying wrist pathology was not investigated.

Cerny et al used a similar scan technique to assess the value of axial distension in the detection of intrinsic ligament and triangular fibrocartilage complex tears in 20 symptomatic subjects [12]. In addition to a widening and improved coverage of the radiocarpal and midcarpal joints, they found a significant increase of the scapholunar distance. Although the LT space width did not increase significantly overall, there was a significant change in width when comparing complete versus partial LT ligament tears. The number of detected partial and complete SL and LT increased with traction, but did not reach statistical significance. These authors suggest that a step-off deformity in Gilula’s carpal arcs during traction might be a highly specific but poorly sensitive sign of SL and/or LT ligament tears.

To study in detail the effects of axial traction to the wrist, Leventhal et al measured the interbone distances on a CT scan in healthy subjects, both at rest and with a 10 kg axial load [13]. They found that the length of the carpus, measured from the radius to third metacarpal, increased on average by 3.3 mm. The distraction was significant at the radiocarpal (radioscaphoid, radiolunate and lunocapitate) and midcarpal joints. These findings are in line with our results. They also observed a radial translation of lunate and triquetrum towards the fixed scaphoid, resulting in a narrowing of the proximal carpal row.

This study has several limitations. Firstly, and most importantly, since the MR exams with and without traction were performed on different patients, a certain degree of selection bias was inevitably introduced. The age and sex of the patients however, were evenly distributed between the two groups. Secondly, although the total number of included patients is higher than in the earlier studies on this subject, the number of cases per subgroup is still rather low and sample were unpaired. This small sample size limits the statistical power and future studies will be needed to confirm our results.

Third, no surgical or arthroscopic correlation was assessed. This study is our next step, but it will require a long delay due to the inclusion of patients concerning cartilage degenerative and osseous traumatic lesions which are not systematically operated. Fourth, we provide neither inter-nor intra-observer variability assessment, since our readings were performed in consensus. Further, the difference in experience between the two readers could limit the validity of our findings. Lastly, the MR exams were performed at 1.5T, whereas 3T is more performant to detect ligament tears [19]. However, despite this limitation, we were able to find significant differences between the subgroups.

In conclusion, opacification score was significantly better for ulnocarpal and CMC III joint spaces with axial traction. A significant difference in radioscaphoid, radiolunate, lunocapitate and ulnocarpal joint space width between coronal T1-images before and during axial traction was systematically discovered in case of ligamentous sports injury and not in degenerative or traumatic subgroups. Indeed, the positive effect of axial traction raises the suspicion of sports ligamentous lesions.
